# Enhanced optical and structural traits of irradiated lead borate glasses via Ce^3+^ and Dy^3+^ ions with studying Radiation shielding performance

**DOI:** 10.1038/s41598-024-73892-w

**Published:** 2024-10-18

**Authors:** O. I. Sallam, Y. S. Rammah, Islam M. Nabil, Ahmed M. A. El-Seidy

**Affiliations:** 1 Glass Lab, Radiation Chemistry Department, National Center for Radiation Research and Technology, Egyptian Atomic Energy Authority (EAEA), Cairo, Egypt; 2 Department of Physics, Faculty of Science, Menoufia University, Shebin El-Koom, Menoufia 32511 Egypt; 3 Pharos University in Alexandria, Canal El Mahmoudia Street, Beside Green Plaza Complex, Alexandria, 21648 Egypt; 4 Physics Department, Faculty of Science, Fayoum University, Fayoum, Egypt; 5 Inorganic Chemistry Department, Advanced Materials Technology & Mineral Resources Research Institute, National Research Centre, El-borough St., P.O. 12622, Dokki, Cairo Egypt

**Keywords:** Lead borate glass, Dysprosium, Cerium, Radiation shielding, Optical properties, XPS, Chemistry, Materials science

## Abstract

Lead borate glass is the best radiation shielding glass when lead is in high concentration. However, it has low transparency after radiation exposure. Radiation decreases transparency due to chemical and physical changes in the glass matrix, such as creating or healing defects in the glass network. The addition of rare earth elements like cerium and dysprosium oxides to lead borate glasses can improve their transparency and durability as radiation shielding barriers. The newly manufactured glasses’ optical absorption, structural, and radiation shielding properties were measured. The optical characteristics of the generated samples were examined to determine the effect of the cerium/dysprosium ratio on the structural alterations, specifically in the presence of bridging oxygen (BO) and non-bridging oxygen (NBO). Incorporating Ce^3+^ results in peaks at 195 nm for borate units, 225 nm for Ce^3+^, and a broadened peak at 393 nm due to overlapping peaks for Ce^3+^ and Ce^4+^ in the UV region. By adding Dy, multiple peaks are observed at 825, 902, 1095, 1275, and 1684 nm, corresponding to the transition from ^6^H_15/2_ ground state to ^6^F_5/2_, ^6^F_7/2_, ^6^F_9/2_, ^6^F_11/2_, and ^6^H_11/2_. The samples were also tested before and after exposure to gamma irradiation from a ^60^Co source at a dose of 75 kGy to assess their stability against radiation. The energy gap value during irradiation shows decreased non-bridging oxygen. The energy gap difference before and after irradiation for the M4 sample shows higher NBO to BO conversion, reducing radiation damage and improving structural stability. Furthermore, X-ray photoelectron spectroscopy was utilized to get insight into the coordination chemistry of the created glass samples. The half-value layer (HVL), radiation protection efficiency (RPE), neutron removal cross-section (FRNCS), mean free path (MFP), mass attenuation coefficients (MAC), and effective atomic numbers (Z_ef_) of the glassy structure were calculated theoretically to assess its radiation shielding qualities. The linear attenuation coefficient order for the prepared samples was M1 > M2 > M3 > M4. The FRNCS values were 0.090, 0.083, 0.081, and 0.079 cm^−1^ for samples M1, M2, M3, and M4, respectively.

## Introduction

Borate glasses are highly influential in different research fields due to their distinctive trait of containing both BO_3_ and BO_4_ units in the borate networks^1^. Modifying these two groups’ proportions can yield novel characteristics suitable for various uses. The BO_4_ and BO_3_ ratio may be modified by adjusting several factors, such as introducing intermediate oxides, adding transition metals (TM) or rare earth elements (RE), utilizing an external radiation source, or applying heat treatment^2^. Borate glasses have exceptional thermal stability, excellent optical properties, a remarkable ability to accommodate rare earth (RE) and transition metal (TM) doping, a low melting temperature, impressive mechanical strength, and superior electrical resistivity^3^.

Borate glasses have poor chemical stability. To address this problem, adding heavy metals to these glasses enhances their polarizability, refractive indices, density, and transparency. Lead oxide can function as an intermediate oxide, which can transform towards a former oxide when present in high quantities, owing to a Pb-O covalent link. However, when it functions as a modifier, this may be attributed to the influence of the Pb-O ionic bond^4^.

Radiation may induce defects in any material. Gamma rays, when passing through a glass matrix with high density, induce the generation of excited electrons from the ground state, capable of migrating inside the glass matrix. The moving electrons can be trapped in defects within the matrix, so mitigating the impact of radiation inside the glass network and impeding the transmission of radiation across the glassy matrix. Regarding the recent progress made in constructing nuclear power plants in different countries to enhance energy generation, it is essential to acknowledge that the presence of ionizing radiation poses a significant risk to workers in this industry, potentially resulting in detrimental effects on living tissues. Glasses can serve as effective radiation shielding barriers, providing protection between the human body and radioactive sources. Glass may be readily produced and molded, exhibits transparency in contrast to concrete, and can withstand cracks that may form in concrete barriers^5^.

Effective radiation shielding is crucial in minimizing the potential harm to employees from radiation since it is contingent upon the radiation’s specific kind and energy level^6^. To reduce the risks associated with radiation, ongoing attempts to improve the characteristics of materials and their ability to block radiation have resulted in the creation of novel compounds for use in radiation shielding applications^7^.

Glass containing heavy metal (HM) oxides, such as PbO, offers excellent shielding against gamma rays. Due to its high density and high effective atomic number of each element of the glass composition, it leads to low relaxation times and half-layer values^8^. Lead glass is often used for radiation shielding purposes. Different varieties of lead glass have been utilized in medical facilities for screens, windows, doors, and walls. Lead glass is commonly used in radiation facilities, hot cells, labs, and radioactive storage stations. It is also employed in fuel development and for many purposes related to nuclear reactors. This is because lead glasses possess the dual properties of transparency and effective attenuation of high-energy photons^9^.

Dysprosium and cerium are elements of the rare earth ions of lanthanide commonly employed in glasses to produce white light devices and characters for lasers in optoelectronics and scintillators. Additionally, they improve the glass’s optical properties^10,11^. Dysprosium and cerium ions exhibit favorable radiation shielding properties of X-rays, neutrons, and γ-rays^12–14^.

This work aims to fabricate shielding lead borate glass with new properties. Doping Ce and Dy ions into lead borate glass is an excellent synergetic tool for combining good optical and structural characteristics for radiation-protective glass even after exposure to radiation. The properties of the prepared glasses have been investigated for this aim.

## Experimental techniques

### Materials and glass preparation method

Alfa chemicals supplied the necessary ingredients for this investigation, which included lead oxide, boric acid, cerium oxide, and dysprosium oxide. Glass samples were produced by the traditional melting method using a high-temperature muffle (Lenton, manufactured in the United Kingdom). The composition of the samples, expressed in mol%, is listed in Table 1. The melting process continued for 60 min at 1100 °C. To achieve consistency in the melted components of each sample, it was necessary to stir all molten specimens twice. The liquefied sample was positioned within warmed stainless-steel frames. The liquefied object was relocated to a secondary furnace at 300 °C to reduce thermal stress. Subsequently, the sample was allowed to cool to the ambient temperature for one day. The collected samples have undergone polishing and categorization into two categories: powder samples and other blocks measuring 1 × 2 cm. The powder samples were utilized for X-ray photoelectron spectroscopy (XPS) studies, whereas the blocks were employed for optical analyses. The produced samples were coded as M-X and individually as M1, M2, M3, and M4, as in Table 1.

**Table 1:** Composition and density of the prepared lead borate glass samples.

Sample	Composition (mol%)				Density (g/cm^3^)
	PbO	B_2_O_3_	Ce_2_O_3_	Dy_2_O_3_	
M1	70	30	0	0	5.469
M2	70	30	0	2	5.061
M3	70	30	2	0	4.946
M4	70	30	2	2	4.729

### Characterization techniques

#### X-ray photoelectron spectroscopy (XPS) studies

The K-ALPHA instrument (Thermo Fisher Scientific, USA) achieved a pressure range of 10^−9^ mbar and a spot size of 400 micrometers for monochromatic X-ray Al K-alpha. Both the broad and narrow spectra exhibited pass energies of 200 electron volts (eV).

#### Density measurements

The density of all glass samples was evaluated using the Archimedes method, employing toluene as the immersion liquid at room temperature according to the following equation:

1 $$\rho ~=~\left( {\frac{{~{W_{air}}}}{{{W_{air}} - ~{W_t}}}} \right){\rho _o}$$

Here, ρ_o_ represents the density of the liquid toluene, which is 0.86 g/cm^3^. W_air_ refers to the weight of the glass when it is in air, whereas W_t_ represents the weight when it is in toluene.

#### Optical studies

To determine the optical measurements, the polished samples, which had dimensions of 1 × 2 cm with 2 ± 0.1 mm of thickness, were analyzed by the UV–Visible/near-IR spectrophotometer of a model V-770 from Japan) and utilized at room temperature. The PMT detector was used to assist with the measurement.

#### Gamma irradiation facility

A Canadian gamma cell of ^60^Co with a dosage rate of 0.966 kGy/h created at the Egyptian Atomic Energy Authority was used to expose the samples to 75 kGy. At the same time, they were situated at room temperature.

#### MCNP simulation

**Fig. 1:**
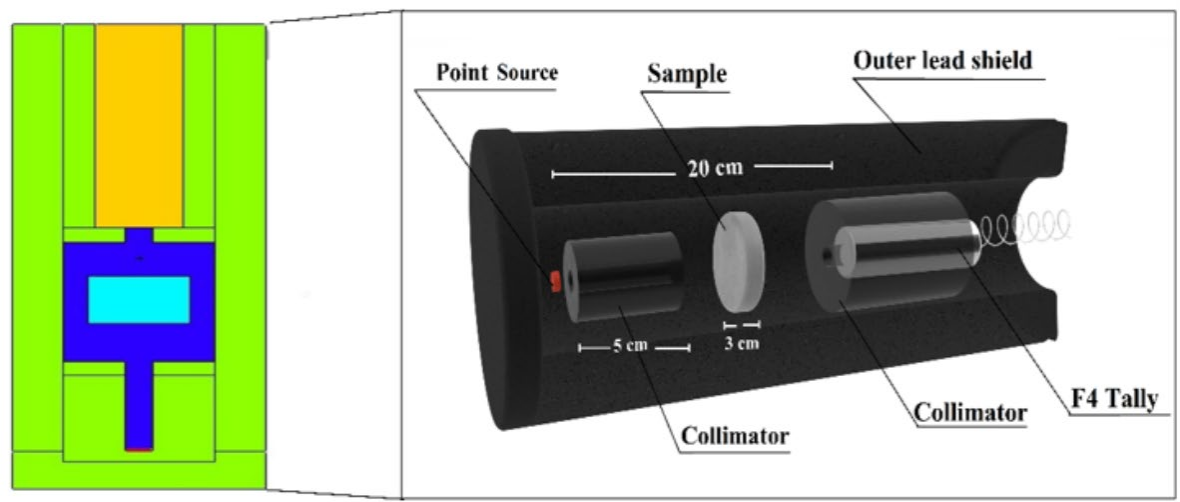
The dynamic view of the radiation attenuation simulation system used for the prepared M-X glass samples.

The code is designed to simulate natural particles using the Monte Carlo method. The MCNP simulation system was utilized to expect the theoretical intensity of γ-rays emitted by γ-point sources. The simulation involved an energetic γ-emitter source within the photon energy range (Pγ) at 0.015 ≤ Pγ ≤ 15 in MeVs. The objective intended to compare the intensity of γ-rays before and following passing through the glass materials being investigated. MCNP codes are commonly favored in research investigations, including radiation shielding and safety, dose calculation, detector design, etc^15–17^. This preference is attributed to an array of advantageous characteristics exhibited by these codes, such as their ability to operate across a broad energy spectrum, flexibility in accommodating various geometrical designs, and rapid calculations. This technique aims to facilitate the movement of electrons, neutrons, and γ-rays, considering several mechanisms of photon interaction. To perform an MCNP simulation, it is necessary to provide precise information in the input file regarding the geometry, source-to-detector distance, source dimensions (SDEF card), and the elemental and chemical composition and densities of the examined concrete samples (material card)^18^. The geometric configuration of the simulation was developed based on a predetermined 2D/3D setup, as represented in Fig. 1. All parameters have been considered consistent with the experimental system. The input files for the MCNP simulation were generated in text lines. The cell involved six distinct components: a radioactive source, 1^st^ and 2^nd^ γ-radiations collimator, a sample with a cubic shape, and a detector. A point source of γ-rays was recognized as an SDEF mono γ-energetic flow for each input file within 0.015 ≤ Pγ ≤ 15 MeV^19,20^. The neutron source has been defined as a californium spectrum, which operates within the E_n_ ≤11 MeV for rapid elimination σ attenuation. The specimens were generated in the form of a cubic layer. Furthermore, the densities and element composition of the examined specimens were recorded in the material card of the text lines. The detector was mounted within a lead collimator designed for the 2nd γ-rays. The command’s Tally computes the sum of values in the range F4:P. At the same time, F4:N calculates the mean length of the incident γ-rays and neutrons released from simulated γ/ neutron radiation sources. An outer shield made of lead was employed to enclose the generated detector, collimators, source, and specimens under investigation. The calculations were performed using a core i5. Multiple NPS (11^7^) attempts were conducted for each file to ensure that random statistical errors remained below 1%. The total runtime for the calculations involving (140) input files was approximately 11 min per run.

For every M-X glass (X = 1, 2, 3, and 4), the incident and transmitted γ-ray photon intensities (I_0_ and I, respectively) were measured. By applying Lambert’s formula, one can determine the linear attenuation coefficient (LAC)^20,21^:

2 $$I={I_o}{e^ - }^{{LACx}},$$

In which the linear attenuation coefficient (LAC) and the thickness (x) of the M-X glass samples are defined. The following is the formula for calculating the mass attenuation coefficient (MAC), a crucial indicator of a material’s property^22^:

3 $$MAC=\frac{{LAC}}{\rho },$$

By adjusting the LAC parameter, the values required to decrease the intensity of incident radiation to half or a tenth of its initial value can be measured. It is possible to determine the HVL and TVL (tenth value layer) in the following way^23,24^:

4 $$HVL=\frac{{ln2}}{{LAC}},$$

5 $$TVL=\frac{{2.303}}{{LAC}},$$

The following equation determines the mean free path (MFP), which is the average track distance the photons move before they collide with the attenuator^25^:

6 $$MFP=\frac{1}{{LAC}},$$

The radiation protection efficiency (RPE) is a crucial metric to consider when assessing the extent to which different shielding materials can reduce radiation. The transfer factor (TF) measures the extent to which photons are passed through the M-X glasses. The calculation of the RPE and TF factors is as follows^26^:

7 $$RPE, \%=\left( {1 - \frac{I}{{{I_o}}}} \right)100$$

8 $${\text{TF}},\% =\left( {\frac{I}{{{I_o}}}} \right)100$$

The incident γ-ray photon intensities with and without material are I and I_o,_ respectively.

The neutron attenuation potential of suggested materials may be determined by calculating the fast neutron removal cross-section (FRNCS) by the equation FRNCS =$\:\sum\:_{i}{W}_{i}$ x ρ. The symbol $\:{W}_{i}\:$presents the partial density, ρ represents the material density, and the subscript i represents the mass cross-section (σ) of the component^27^. The half-value layer (HVL_FRNCS_) was determined using the formula. $\:{HVL}_{\text{F}\text{R}\text{N}\text{C}\text{S}}\:=\:\frac{ln2}{FRNCS}$, and the relaxation length (λ_FRNCS_) was calculated as $\:\frac{1}{FRNCS}$^28^.

#### Phy-X software

The PhX software is an online tool that calculates various shielding and attenuation factors for different material compositions^29,30^. The relative differences (φ in %) were determined by comparing the data received from PhX and MCNP in the following manner^31^:

9 $$\varphi \left( \% \right)=\left| {\frac{{MCNP - PhX}}{{MCNP}}} \right| \times {\text{1}}00$$

## Results and discussion

### X-ray photoelectron spectroscopy (XPS)

**Fig. 2:**
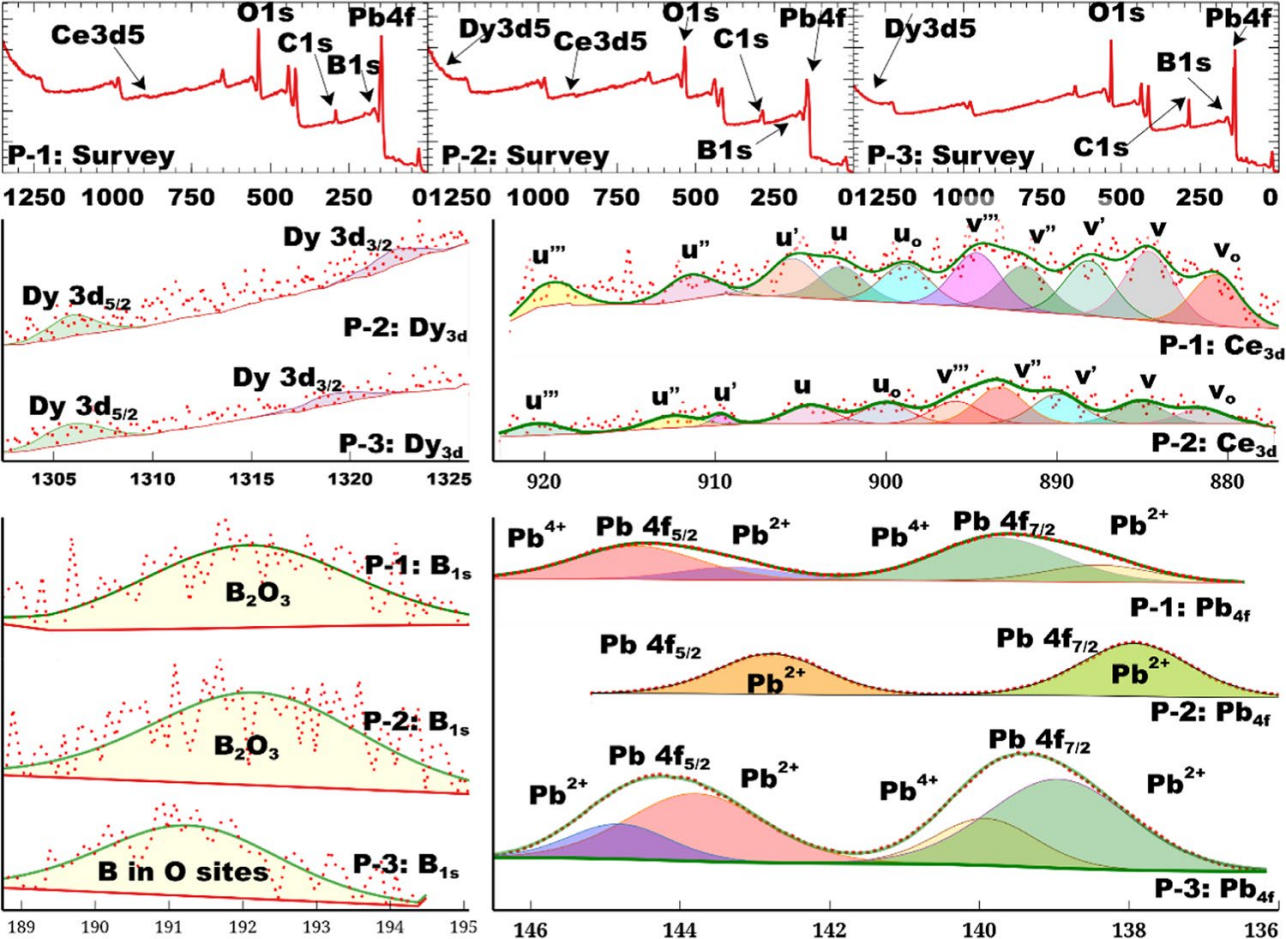
XPS spectra of prepared glass samples.

The XPS survey spectra of M3 (P-1), M4 (P-2), and M2 (P-3) samples are shown in Fig. 2. The survey spectra of glasses show O1s, C1s, B1s, and Pb4f, indicating the presence of oxygen, carbon, boron, and lead as the basic components. The survey spectra of M3 (P-1) and M2 (P-3) showed cerium (Ce3d_5_) and dysprosium (Dy3d_5_), respectively, while that of M4 (P-2) showed the presence of both dysprosium (Dy3d_5_) and cerium (Ce3d_5_). The presence of C-1s in all spectra is owing to the adsorption on the surface of the glassy sample or as a residue. The fitted curves agreed with the experimental curves in all HRs (high-resolution spectra). The HRs of B1s show no peaks around 188.7 eV, suggesting no B^0^ exists^32^. The HRs of M3 (P-1) and M4 (P-2) show peaks in 192.06–192.18 eV, while that of P-3 is at 191.28 eV, corresponding to B_2_O_3_ and substitutional B that occupy O sites, respectively^33,34^. The HRs of Dy show two peaks in 1302.79-1305.88 eV and 1319.16-1322.10 eV regions, which may be assigned to Dy 3d_5/2_ and Dy 3d_3/2_, respectively^35–37^. The doublets in 137.93-138.94 and 142.78-143.79 eV regions in the HRs of Pb 4f may be allotted to Pb 4f_7/2_ and Pb 4f_5/2_, respectively, for Pb (II) oxidation state, while the doublets in 139.75-139.92 and 144.59-144.82 eV regions in the spectra of M3 (P-1) and M4 (P-2) may be allocated to Pb 4f_7/2_ and Pb 4f_5/2_, respectively for Pb (IV) oxidation state^38–40^. The % Pb^2+^ and % Pb^4+^ were calculated using peak areas. The spectra of M3 (P-1) and M2 (P-3) showed % Pb^+2^ were % 24.97 and %72.60, respectively, while % Pb^+4^ was %75.03 and % 27.40, respectively. The HRs of Ce show a set of ten peaks indicating the presence of Ce in + 3 (v_0_, v′, u_0_ and u′) and + 4 oxidation states (v, v′′, v′′′, u, u′ and u′′′)^41^ Ce 3d spectrum shows five spin-orbit doublets from Ce 3d_5/2_ (v) and Ce 3d_3/2_ (u). u′′′ may be regarded as Ce^4+^ fingerprint^42^. The % Ce^3+^/Ce^4+^ was calculated that, giving the % Ce^3+^/Ce^4+^ for P-1 and P-2 were found to be %66.25 and 59.18, respectively.

### Optical studies

**Fig. 3:**
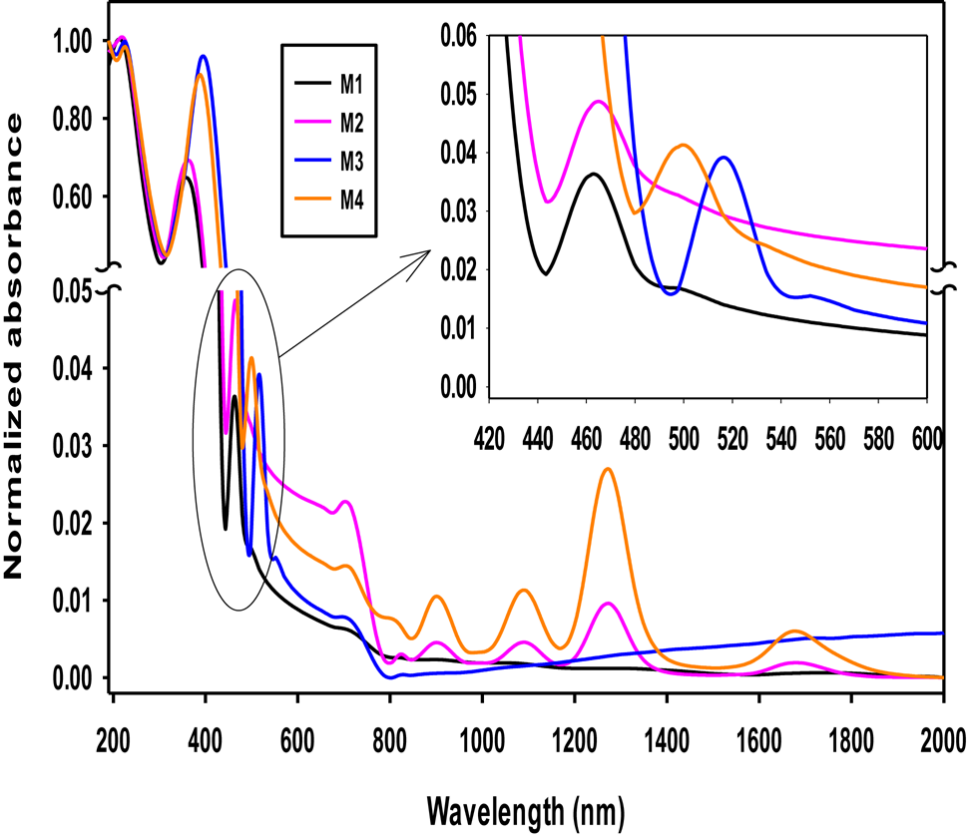
Normalized absorbance spectra of lead borate glasses (undoped, doped with Dy or Ce, and coped with Dy-Ce).

Figure 3 illustrates the absorption spectra of undoped lead borate glasses, singly doped with Dy, Ce, and co-doped with Dy-Ce. All samples show a broad peak in the 190–231 nm range related to borate host glass^43^. Undoped sample (M1) and singly doped with Ce (M3) give nearly similar spectra with few differences observed at the peak of 210 nm that were found only in the case of M1 and M2 but splitting into two peaks in the case of incorporation of Ce^3+^ as shown at M3 and M4 samples to give peaks at 195 nm for borate units and 225 nm for Ce^3+^ only for 4 F→5d transition (^2^F_5/2_→^5^D_1_)^44^. The observed shift in borate unit peaks after adding Ce^3+^ may be related to the role of cerium oxide addition in converting some BO_3_ units to BO_4_ and increasing the numbers of non-bridging oxygens (NBOs). A second change in the peaks has been observed after adding Ce_2_O_3_ to M1; in the case of M1 and M2 samples, the peak at 360 nm is broadened with higher intensity to be at 393 nm in case of M3 and M4 due to overlapping of two peaks for Ce^3+^ and Ce^4+^ in the UV region due to the 4f/5d transition of Ce^3+^ ions beside the Ce^4+^/Ce^3+^ charge transfer absorption peak of Ce^4+^ ions^45,46^.

By adding Dy^3+^ ions to lead borate glass system in case M2 and M4, several peaks are observed at 825, 902, 1095, 1275, and 1684 nm corresponding to the transition from 6H15/2 ground state to the transition states ^6^F_5/2_, ^6^F_7/2_, ^6^F_9/2_, ^6^F_11/2_ and ^6^H_11/2_; respectively. The hypersensitive transition was observed at 1275 nm due to ^6^F_11/2_^47^. The intensity of the peaks for M2 and M4 changes due to the conversion of BO_3_ units (sp^2^) to BO_4_ units (sp^3^), which is highly stable with more NBOs. By adding Dy into the lead borate system, the intensity increases at 390 nm from M1 to M2 and grows with a shift in the case of M4.

**Fig. 4:**
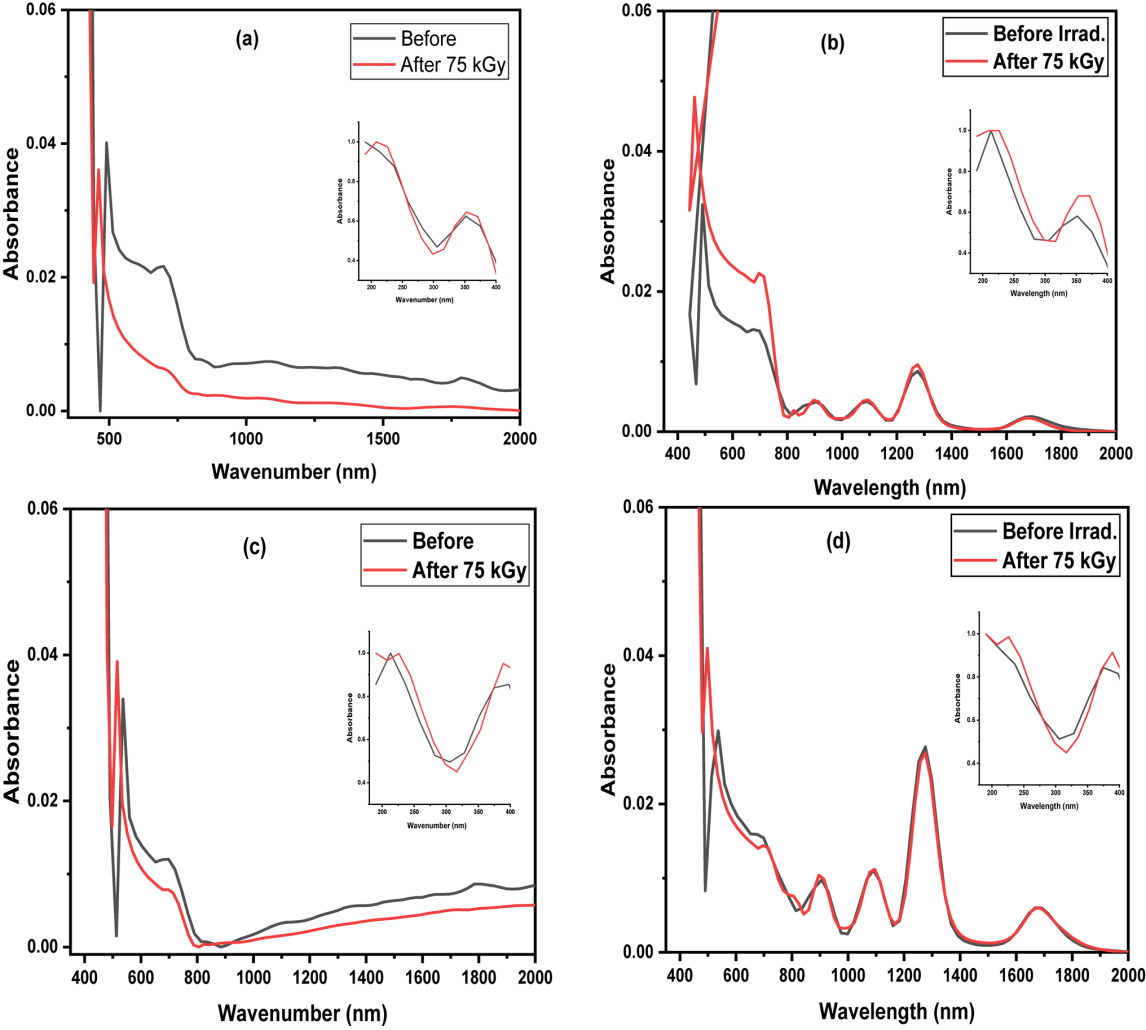
Normalized absorbance spectra of lead borate glasses before and after irradiation with 75 kGy (**a**) M1 (**b**) M2 (**c**) M3 and (**d**) M4.

**Fig. 5:**
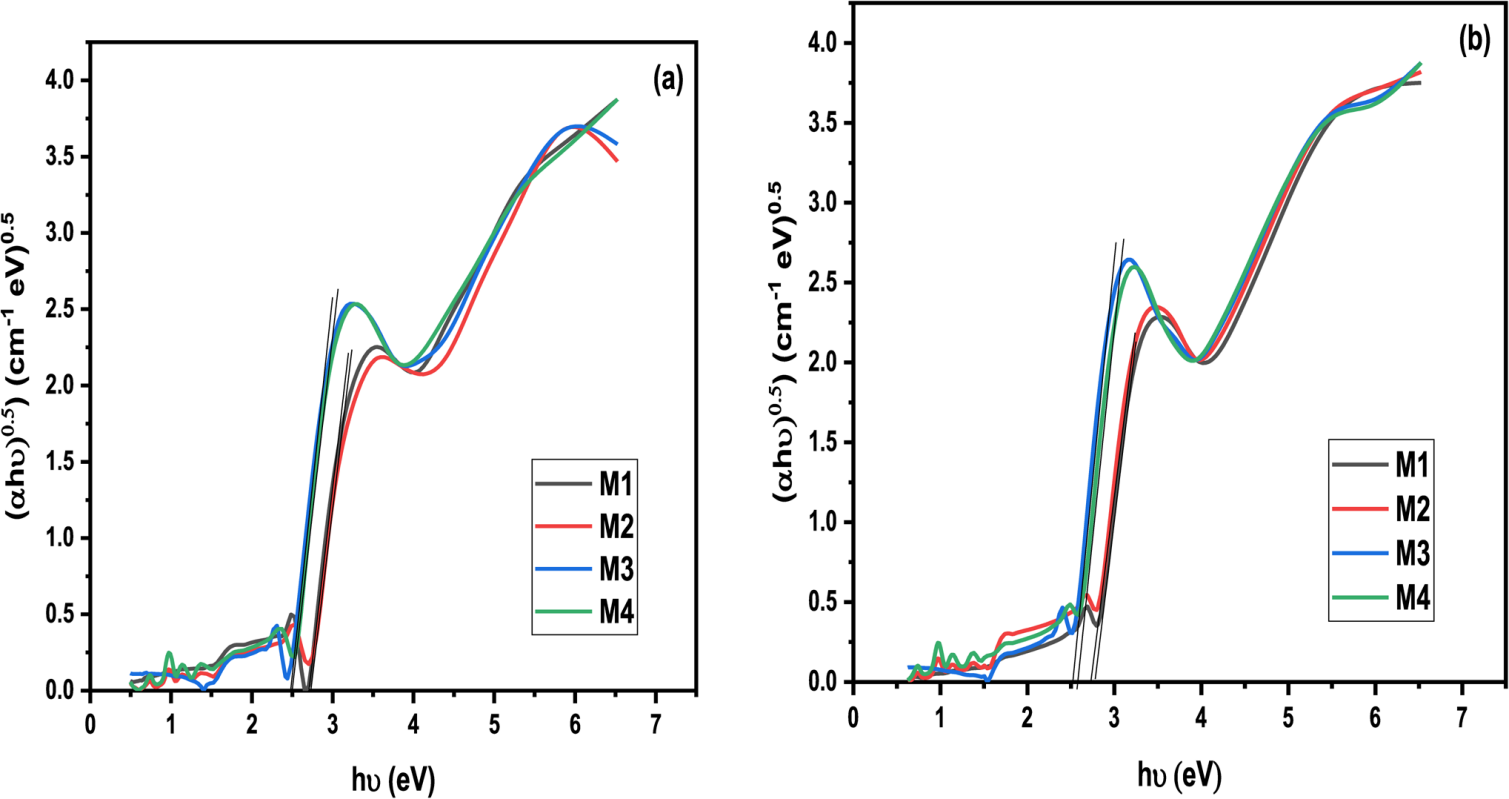
Tauc plots for all samples (**a**) before and (**b**) after irradiation by 75 kGy.

No new peaks were observed after exposure to gamma radiation dosages of 75 kGy. However, there was a slight change in the intensity of the existing peaks, indicating the glass structure’s resilience against radiation. This was particularly evident in the case of M4, where the curves remained practically unchanged before and after irradiation, as shown in Fig. 4. When cerium and dysprosium ions are added, the energy gap values of the lead borate system provide insights into the changes occurring within the energy levels. These findings are presented in Fig. 5a, b; Table 2. The incident photon energy determines the indirect band gap energy and can be obtained using the Tauc’s equation^48^.

**Table 2:** Energy gap values for all samples before and after γ-irradiation by 75 kGy.

Symbol ID	Energy gap value (E_g_)		ΔE_g_
	Before	After	
M1	2.7394 ± 0.0021	2.7702 ± 0.0035	0.0308
M2	2.7103 ± 0.0031	2.7206 ± 0.0032	0.0103
M3	2.5205 ± 0.0024	2.5702 ± 0.0028	0.0497
M4	2.4900 ± 0.0016	2.5906 ± 0.0029	0.1006

10 $${\left( {\alpha h\upsilon } \right)^{\frac{1}{n}}}=A\left( {h\upsilon - {E_g}} \right)$$

When *n* = 2, if an indirect transition occurs within the amorphous matrix, A is regarded as a constant. The results indicate that adding Dy, Ce, and Dy-Ce decreases energy gap values. This suggests the generation of defects that can effectively capture more free electrons, raising the likelihood of forming non-bridging oxygen atoms (NBOs) and enhancing the optical properties^49^. An increase in the energy gap value following irradiation typically leads to a decrease in non-bridging oxygen, increasing the structure’s compactness due to the higher presence of its bridging oxygen. By calculating the difference between the energy gap after irradiation and before, it was observed that the most significant difference in energy gap values was for the M4 sample (lead borate glass doped with Dy-Ce), where higher conversion from NBO to BO that reflects the more compact structure. This implies that the M4 sample exhibits lower susceptibility to radiation-induced damage, enhancing the sample’s structural stability.

Furthermore, gamma rays have a significant influence on increasing E_g_ values. Therefore, it can be demonstrated that subjecting glass samples to gamma rays leads to the repair of flaws by the conversion of non-bridging oxygen into bridging oxygen. As the radiation exposure increases, the number of defects inside the glass matrix undergoes healing^50^. The Urbach energy (E_u_) can be determined by analyzing the slope of the straight line obtained from the relationship between (hυ) and (ln α) in the absorption figure, as described by equation^51^.

11 $$ln\left( \alpha \right)=ln\left( {{\alpha _o}} \right)+\frac{{h\upsilon }}{{{E_u}}}$$

**Fig. 6:**
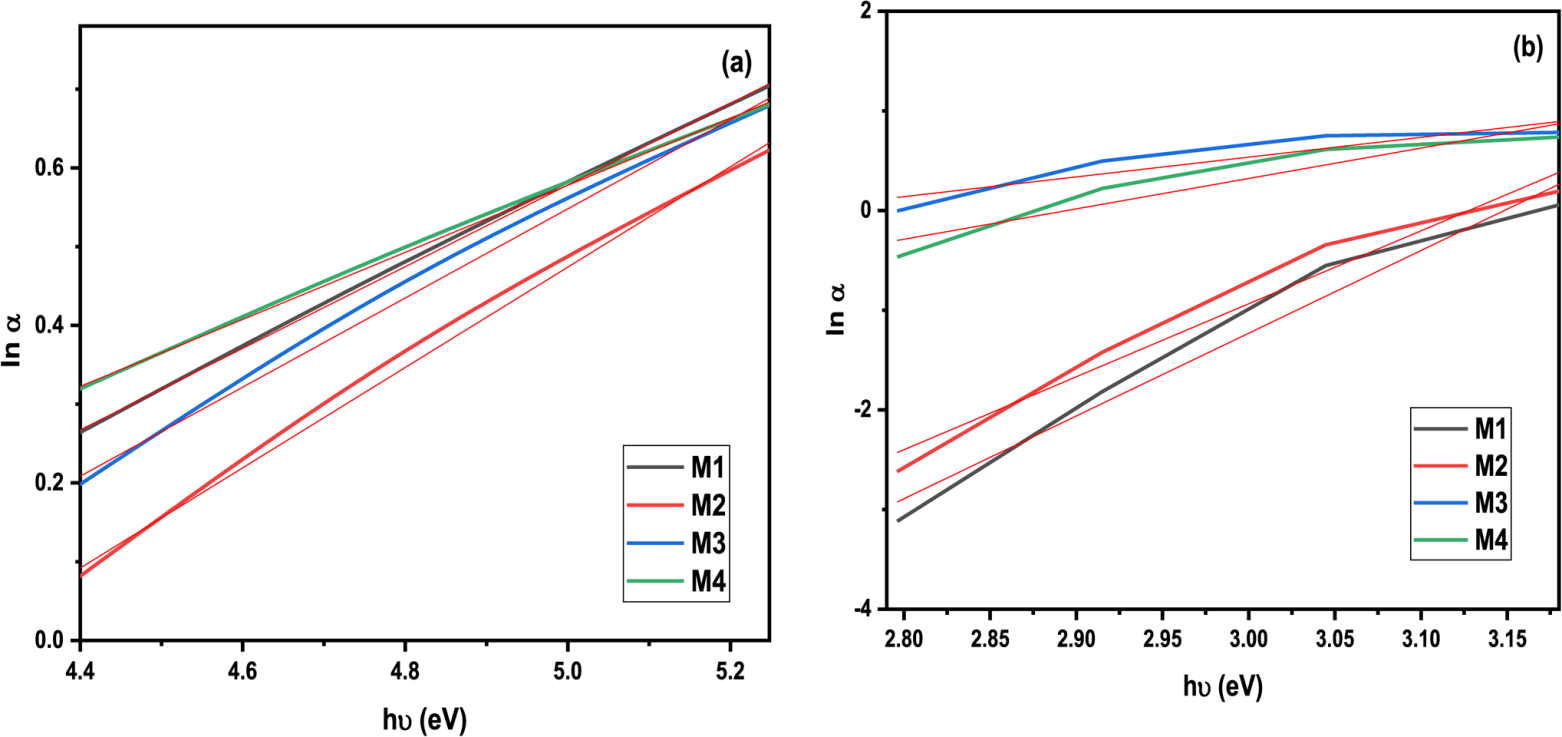
Urbach energy values for all samples (**a**) before and (**b**) after irradiated with 75 kGy.

The Urbach energy values for the prepared system facilitate tracking changes in the disorder characteristics of the glassy network upon the addition of Dy, Ce, and Dy-Ce oxides. According to Fig. 6; Table 3, the network disorder is significantly higher in the case of M4. This indicates a rise in non-bridging oxygen (NBO) atoms and the depolymerization of the glassy structure, resulting in a less compact glass^26^.

**Table 3:** Urbach energy values for all samples before and after irradiation with 75 kGy.

Symbol ID	Urbach gap value (E_u_)		ΔE_u_
	Before	After	
M1	1.9285 ± 0.0086	0.1204 ± 0.0122	1.8081
M2	1.5701 ± 0.0106	0.1366 ± 0.0095	1.4335
M3	1.7669 ± 0.0089	0.5043 ± 0.0098	1.2626
M4	2.3492 ± 0.009	0.3281 ± 0.0101	2.0211

It was noted that the Urbach energy values drop following irradiation, indicating increased stability due to a reduction in the degree of disorder. Furthermore, M4 exhibits the most significant decrease in the disorder caused by radiation.

### Gamma-ray shielding properties

**Fig. 7:**
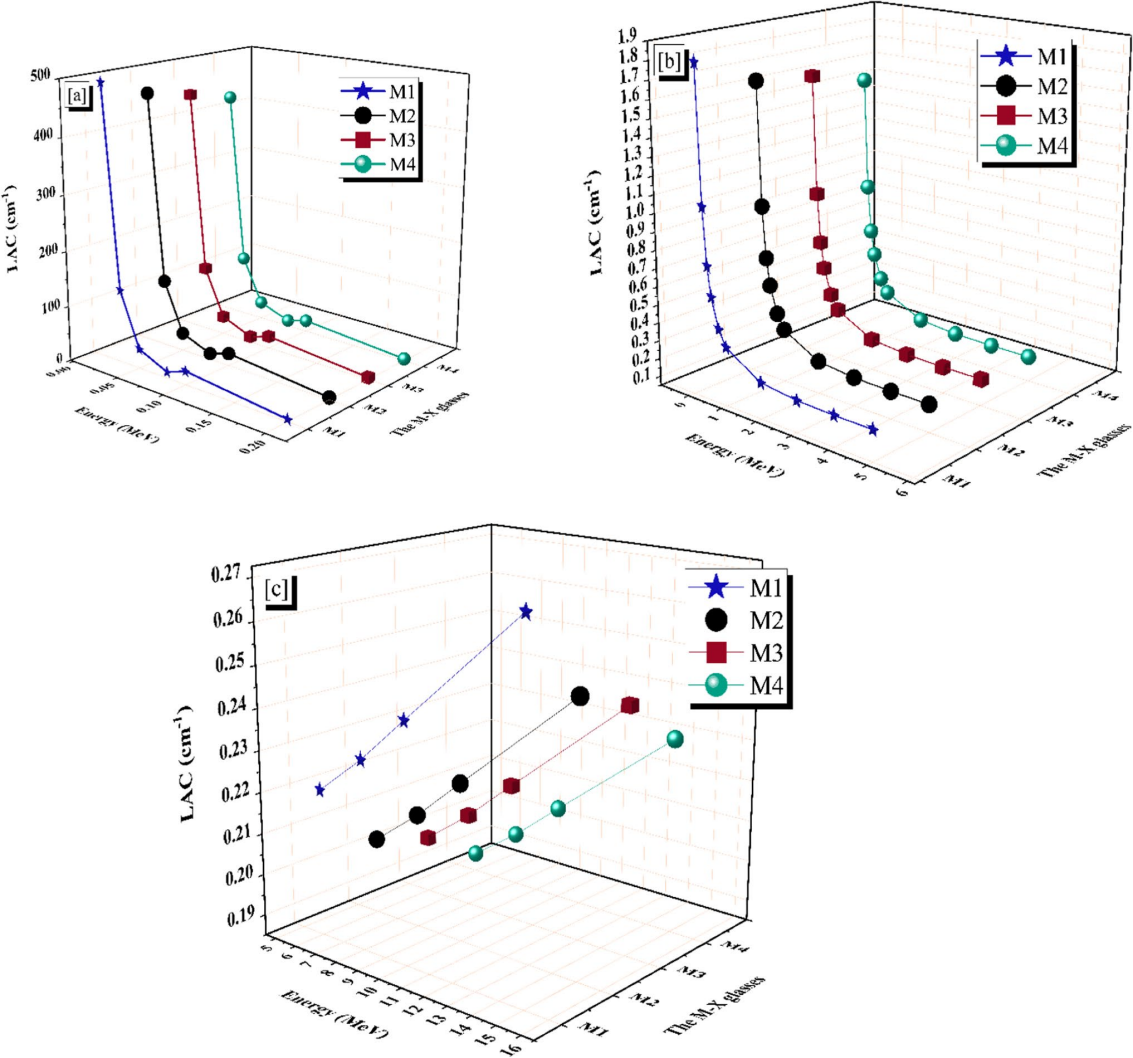
Influence of gamma-ray energy on linear attenuation coefficient of (**a**) photo electric, (**b**) compton scattering, and (**c**) pair production for the M-X glass samples.

Figure 7a–c depicts the LAC (Linear Attenuation Coefficient) of the four M-X glass samples produced. Table 4 displays the LAC values obtained from utilizing MCNP code and PhX software inside the γ-energy range of 0.015 to 15 MeV. The simulated values of LAC are reasonably consistent with the values estimated by PhX, with a maximum φ of 4.957%. The *LAC* values drop from 491.429 to 0.271 cm^−1^ for M1, from 458.103 to 0.250 cm^−1^ for M2, from 440.423 to 0.244 cm^−1^ for M3, and from 419.991 to 0.232 cm^−1^ for M4 sample at γ-energy range (Pγ) at 0.015 ≤ Pγ ≤ 15 MeV.

**Table 4:** The linear attenuation coefficient (*µ*), which obtained using MCNP and PhX for the prepared M-X samples.

Energy, (MeV)	The linear attenuation (LAC, cm^−1^)											
	M1			M2			M3			M4		
	PhX	MCNP	φ %	PhX	MCNP	φ %	PhX	MCNP	φ %	PhX	MCNP	φ %
0.015	501.2813	491.4291	2.0048	463.2722	458.1026	1.1285	449.4738	440.4233	2.0550	427.9645	419.9906	1.8986
0.03	136.1305	132.3763	2.8360	123.7823	120.6753	2.5747	120.8175	117.4046	2.9070	113.2511	110.2890	2.6858
0.05	36.2198	35.1949	2.9121	32.9199	32.1730	2.3215	33.9982	33.0128	2.9850	31.8175	30.9636	2.7578
0.08	10.9987	10.8844	1.0506	10.8425	10.7332	1.0181	10.2936	10.1839	1.0769	10.3987	10.2963	0.9950
0.1	25.0047	24.5730	1.7568	22.8056	22.4325	1.6632	22.2518	21.8582	1.8008	20.9206	20.5782	1.6637
0.2	4.5933	4.4072	4.2238	4.1879	4.0205	4.1642	4.0879	3.9182	4.3295	3.8435	3.6957	4.0001
0.3	1.9103	1.8222	4.8357	1.7446	1.6632	4.8953	1.7034	1.6229	4.9567	1.6050	1.5347	4.5795
0.4	1.1337	1.0871	4.2803	1.0375	0.9941	4.3671	1.0132	0.9706	4.3874	0.9570	0.9197	4.0535
0.5	0.8078	0.7794	3.6423	0.7407	0.7138	3.7617	0.7234	0.6974	3.7335	0.6848	0.6620	3.4494
0.6	0.6375	0.6180	3.1633	0.5854	0.5668	3.2817	0.5718	0.5538	3.2425	0.5423	0.5265	2.9958
0.8	0.4664	0.4569	2.0928	0.4292	0.4198	2.2334	0.4193	0.4105	2.1452	0.3986	0.3909	1.9820
1	0.3803	0.3707	2.5745	0.3504	0.3415	2.5868	0.3423	0.3335	2.6389	0.3259	0.3182	2.4381
2	0.2498	0.2471	1.1077	0.2306	0.2281	1.0923	0.2253	0.2228	1.1354	0.2149	0.2127	1.0490
3	0.2247	0.2230	0.7297	0.2074	0.2059	0.7252	0.2026	0.2011	0.7479	0.1932	0.1919	0.6910
4	0.2181	0.2170	0.5074	0.2014	0.2002	0.5511	0.1967	0.1957	0.5201	0.1876	0.1867	0.4805
5	0.2183	0.2174	0.4068	0.2015	0.2007	0.3877	0.1969	0.1960	0.4169	0.1876	0.1869	0.3852
6	0.2214	0.2206	0.3421	0.2043	0.2036	0.3574	0.1997	0.1990	0.3507	0.1902	0.1896	0.3240
8	0.2312	0.2306	0.2585	0.2134	0.2128	0.2611	0.2085	0.2080	0.2650	0.1986	0.1981	0.2448
10	0.2427	0.2422	0.2343	0.2240	0.2235	0.2519	0.2189	0.2184	0.2402	0.2085	0.2080	0.2219
15	0.2712	0.2707	0.1675	0.2502	0.2498	0.1576	0.2445	0.2441	0.1717	0.2327	0.2324	0.1586

Figure 7a demonstrates a significant decrease in the LAC values of all the manufactured M-X glass samples due to the photo-electric effect (PEE) interaction, which has altered the σ (cross-section) with Z_4_.P_γ_^−4:−5^^52^. As a result, the interaction cross-section reduces significantly when the Pγ values are enriched, leading to a decrease in the photo-electric effect (PEE). The applied Pγ values, 0.015 ≤ Pγ ≤ 0.2 MeV sources have increased a tough exponential falling propensity from 491.429 to 4.407 cm^−1^ for M1, from 458.103 to 4.021 cm^−1^ for M2, from 440.423 to 3.918 cm^−1^ for M3, and from 419.991 to 3.696 cm^−1^ for M4 sample.

Figure 7b demonstrates that the simulated LAC values within the γ-photon range of 0.3 to 5 MeV experience a rapid decrease following an exponential pattern. This reduction is attributed to the increased concentration of Pγ above 0.2 MeV. The exponential drop is attributed to the interplay between Compton phenomena (COM) and the variations in cross-section generated by Z/Pγ^−1^^53^. The reduced tendency of photons with higher energy to interact with the atoms of the substance is due to their incredible velocity.

Consequently, as energy levels rise, the probability of γ-absorption decreases, whereas the probability of photon dispersion increases^54^. The rise in Pγ values was associated with a gradual decline in the σ due to reductions in the number of γ-electron interactions, subsequently leading to a gradual fall in the LAC values. The drop in LAC was from 1.822 to 0.217 cm^−1^ for M1, from 1.663 to 0.201 cm^−1^ for M2, from 1.623 to 0.196 cm^−1^ for M3, and from 1.535 to 0.232 cm^−1^ for M4 glassy sample with levitation the Pγ at 0.300 ≤ Pγ ≤ 5 MeV, respectively.

Also, there is a minor increment due to the pair production (PPE) interaction with σ changes with Z^2^. ${P}_{\gamma\:}^{-2}$^55^ at energy more than 5 MeV, as seen in Fig. 7c. The LAC were from 0.221 to 0.271 cm^−1^ for M1, from 0.204 to 0.250 cm^−1^ for M2, from 0.199 to 0.244 cm^−1^ for M3_,_ and from 0.190 to 0.232 cm^−1^ for M4 glass sample with raising the Pγ values between 6 ≤ Pγ ≤ 15 MeV.

Based on the previous results, M1 shows significantly higher LAC values due to the higher atomic number (Pb = 82) and high samples’ densities (5.469 g.cm^−3^) with high compactness. The LACs of the prepared samples decrease by adding dopants that enter inside the glass network through the interstitial vacancies, making the network more open, which results in decreasing the density of the glass samples. The samples M2 and M3, which had the doping of Dy and Ce, respectively, come in the second and third order in terms of the LAC.

**Fig. 8:**
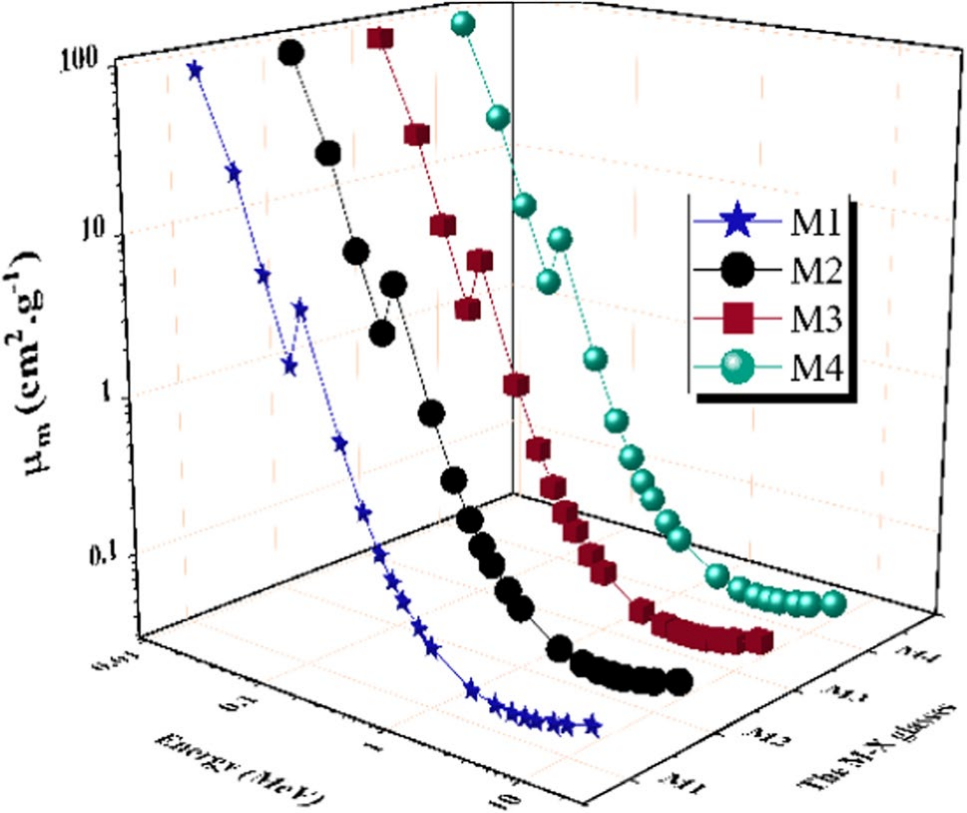
The mass attenuation coefficients (*µ*_*m*_) vs. the photon energy for the prepared M-X glass samples.

The M-X glasses’ mass attenuation coefficient (µm) exhibits comparable behavior to LAC, particularly in the PEE and COM zones. The MAC were varied from 89.844 cm^2^ g^−1^ to 0.049 cm^2^ g^−1^ for M1, from 90.509 cm^2^ g^−1^ to 0. 049 cm^2^ g^−1^ for M2, from 89.039 cm^2^ g^−1^ to 0.049 cm^2^ g^−1^ for M3, and from 88.804 cm^2^ g^−1^ to 0.049 cm^2^ g^−1^ for M4 glass sample as realized in Fig. 8.

**Fig. 9:**
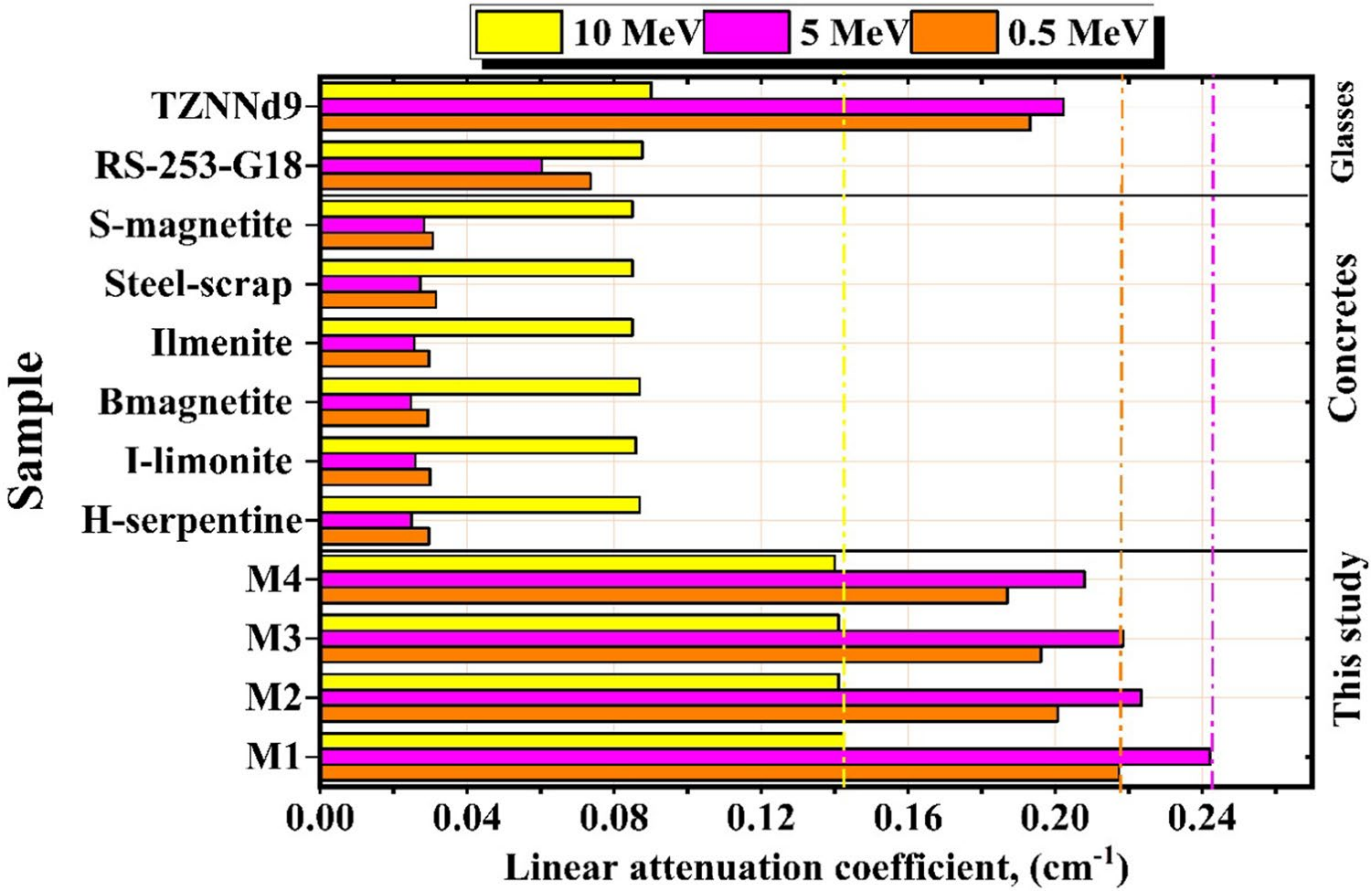
The linear attenuation coefficient for the M-X glass samples with reference concretes and glasses.

Figure 9 compares the LAC values for M-X glasses, commercial concrete (I-limonite, H-serpentine, Ilmenite, B-magnetite, S-magnetite concretes, and S-scrap), and many common glasses (TZNNd9, otherwise RS-253-G18) at selected energies of 0.5, 5, and 10 MeV^54,56^. The LAC values of M-X glasses are higher than those of glass and concretes at 0.5, 5, and 10 MeV.

**Fig. 10:**
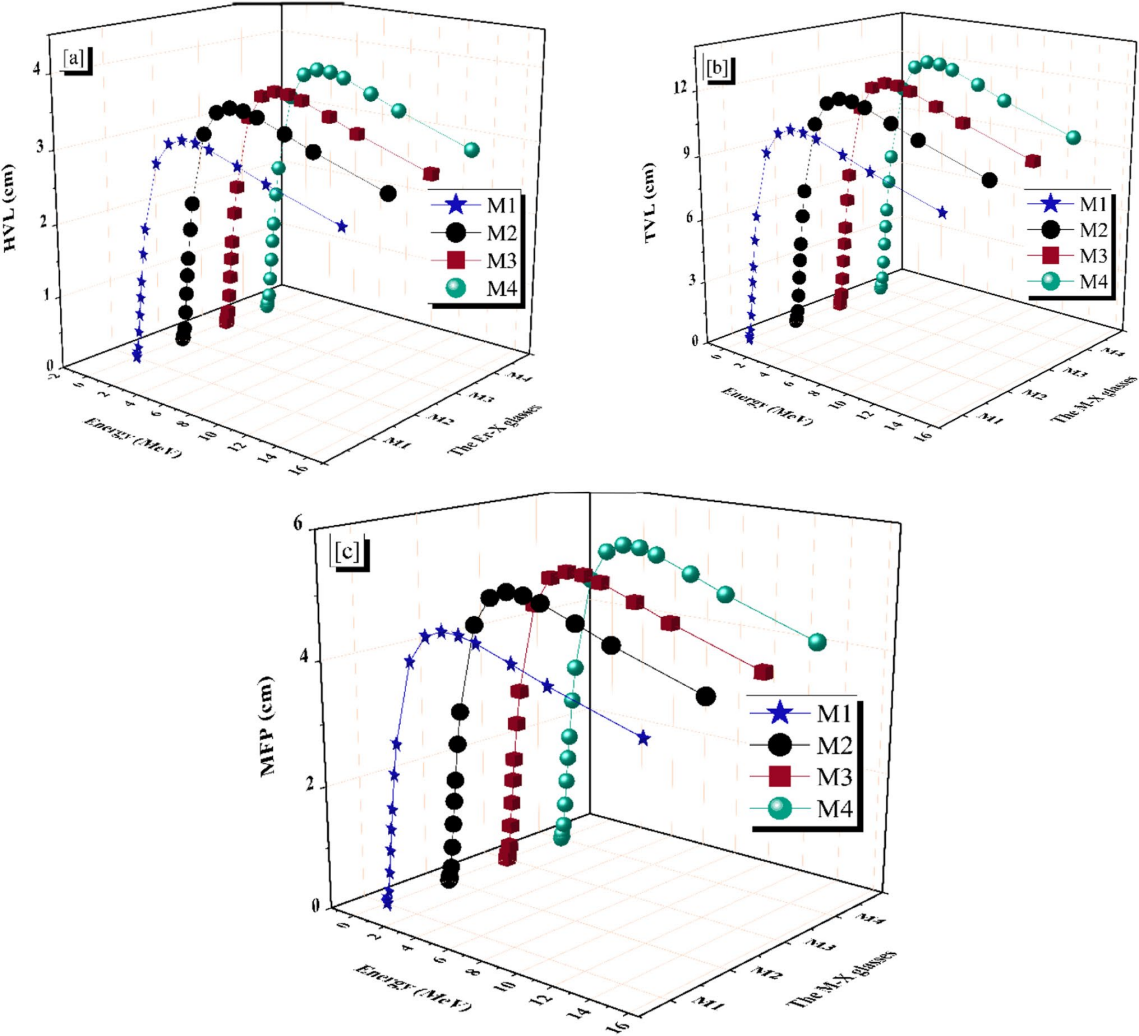
(**a**) The half value layer (HVL), (**b**) The tenth value layer (TVL), and (**c**) The mean free path (MFP) for the prepared glass M-X samples vs. the photon energy.

Common metrics for radiation shielding efficiency include the half- and tenth-value layers (HVL, TVL) and the mean free path (MFP). A lower value for either parameter typically results in a stronger radiation shielding concert for assumed Pγ because radiation is dampened as it passes a narrower zone^57^. The results of using Eqs. (4–6) to calculate HVL, TVL, and MFP are shown in Fig. 10a–c. The HVL of the M-X glass samples that were studied rose as the LAC values fell. At 0.015 ≤ Pγ ≤ 15 MeV, the HVL values for M1, M2, M3, and M4 glass samples increased from 0.001 cm to 2.561 cm, 2.774 cm, 2.839 cm, and 2.983 cm, respectively, as shown in Fig. 10a.

The TVL and MFP values yield the equivalent tendency as the HVL, as seen in Fig. 10b, c. The MFP values range from 0.002 cm to 3.694 cm for M1, from 0.002 cm to 4.003 cm for M2, from 0.002 cm to 4.096 cm for M3, and from 0.002 cm to 4.303 cm for the M4 glass sample, respectively.

Conclusions drawn from the data show that the additives to lead borate glass affected the HVL, TVL, and MFP values. Thus, the M4 glass sample had the most significant values for HVL, TVL, and MFP, whereas the M1 glass sample had the lowest values^58^.

**Fig. 11:**
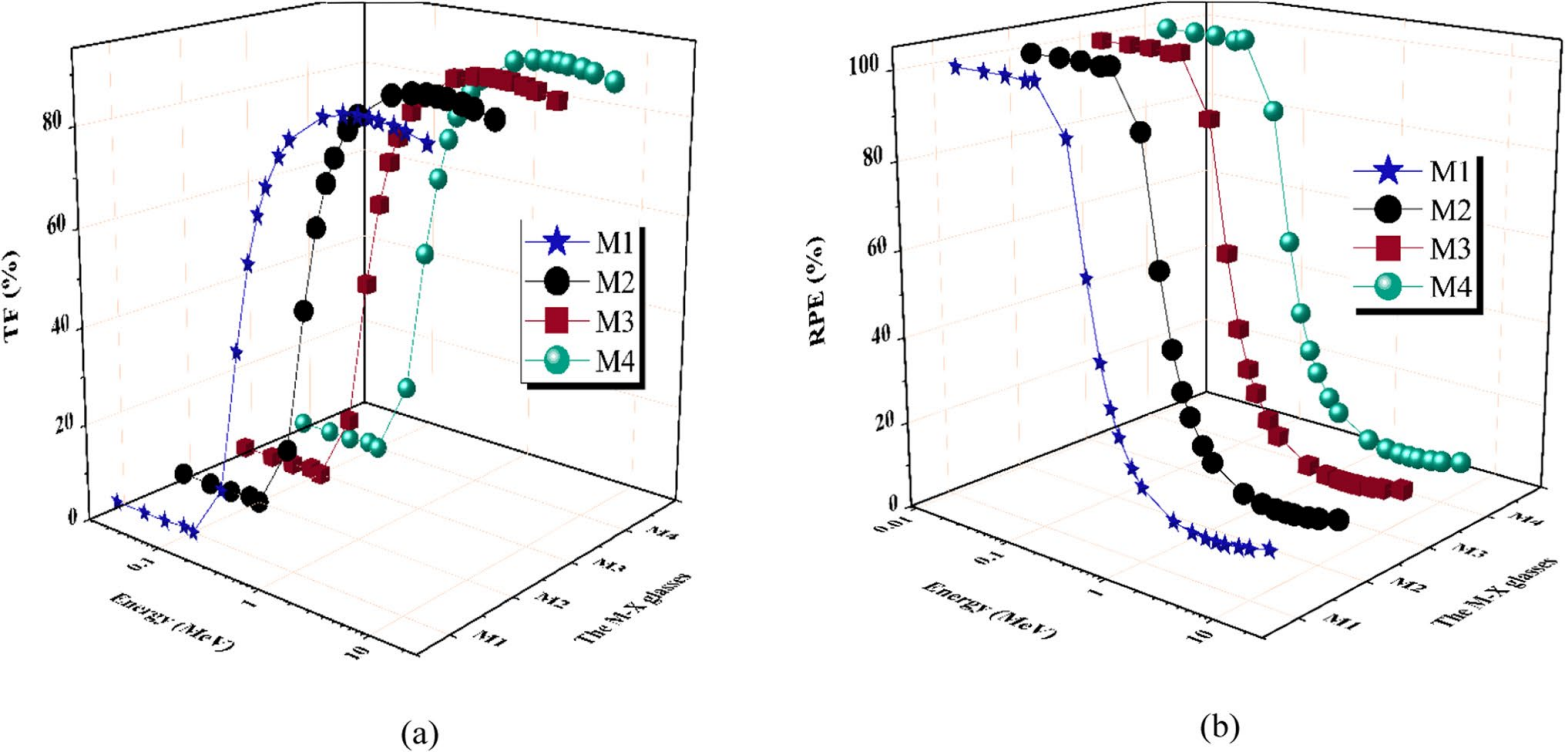
(**a**) The transfer factor (TF) and (**b**) the radiation protection efficiency (RPE) for the prepared M-X glass samples vs. photon energy.

Figure 11a displays the transfer factor of the M-X glasses. Furthermore, the RPE (radiation protection Efficiency) of the manufactured M-X glass samples was influenced by the energy of the γ-rays and the concentration and elements used for doping, as depicted in Fig. 11b. The data indicates that the RPE values approach 100% when the gamma-ray energy is between 0.015 MeV and 0.100 MeV. As the energy of the γ-photons grew, the ability of the photons to penetrate likewise increased, resulting in a notable reduction in the levels of RPE^59–61^. Hence, when the energy of γ-photons increases, the interactions between photons and electrons in the produced glasses decrease. The quantity of dispersed photons escalates when the interaction between photons and electrons is diminished, adversely impacting the RPE for the M-X glass samples. The RPE values decreased from about 100% at 0.100 MeV for all M-X glass samples to 88.959%, 86.605%, 85.902%, and 84.242% for M-X glass samples with X values of 1, 2, 3, and 4, respectively, at a γ-ray energy of 0.200 MeV. The RPE values decreased from 59.791 to 12.659% for M1, from 56.465 to 11.743% for M2, from 55.579 to 11.491% for M3, and from 53.577 to 10.969% for M4 glass sample as the energy values increased from 0.300 ≤ Pγ ≤ 15 MeV, respectively. The RPE takes the order: M1 ≥ M2 ≥ M3 ≥ M4. The results demonstrate that the M-X glass samples have a high level of shielding effectiveness in the energy range of 0.015 ≤ Pγ ≤ 0.300 MeV.

**Fig. 12:**
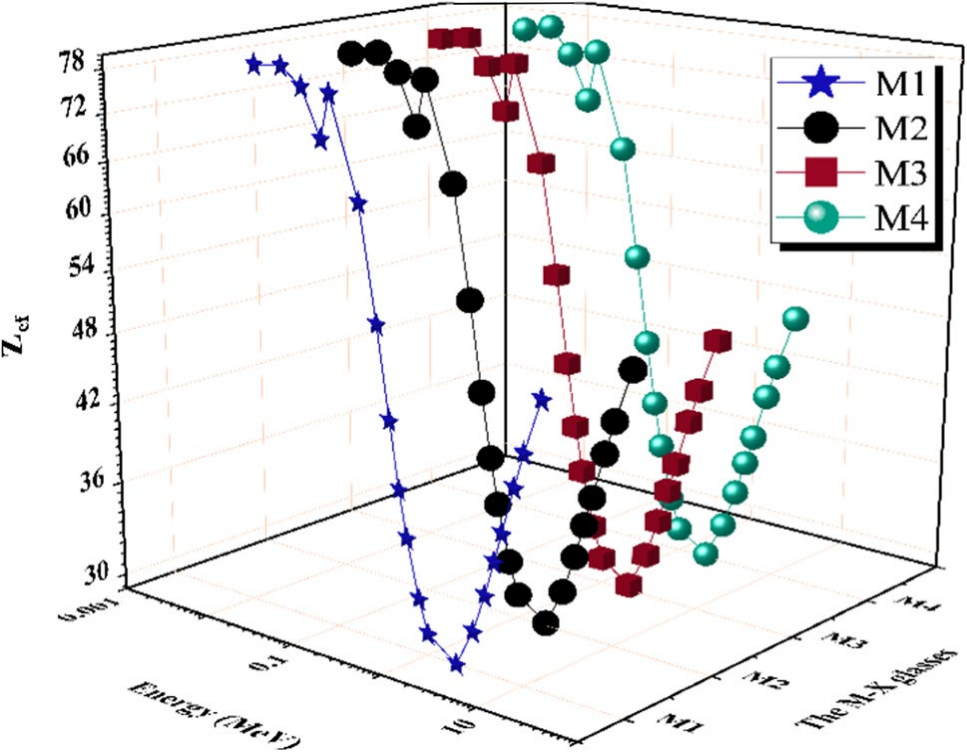
The effective atomic number (Zef) for the prepared M-X glass samples vs. photon energy.

A sample’s effective atomic number (Z_ef_) is a vital metric with several practical applications in modern technology, physics, and engineering. Z_ef_ quantifies the distinct properties of a substance. Figure 12 illustrates the correlation between the Z_ef_ and the Pγ for the analyzed substances. Elevations in Z_ef_ value typically suggest heightened radiation interaction, mainly via the COM effect and PEE, within a specific substance. Therefore, highly Z_ef_-value materials are more effective at blocking high-energy radiation^62,63^. This demonstrates that the effectiveness of various materials in attenuating radiation can vary depending on the energy of the radiation, with materials that are more effective in attenuating radiation with higher MeV and lower MeV being required. The Z_ef_ range for the created M-X glass samples ranged as follows: 79.631–49.007 for M1, 78.920–48.966 for M2, 78.898–49.062 for M3, and 78.197–48.704 for M4. The M1 glass has the highest Z_ef_ values among the four M-X series glasses. The glass M1 sample exhibits the highest Zef values within the energy range of 0.015 to 5 MeV, primarily due to its high density compared with other prepared samples. The glass M3 sample exhibits the highest Z_ef_ values within the energy range of 0.500 to 15 MeV due to the content of the Ce element.

**Fig. 13:**
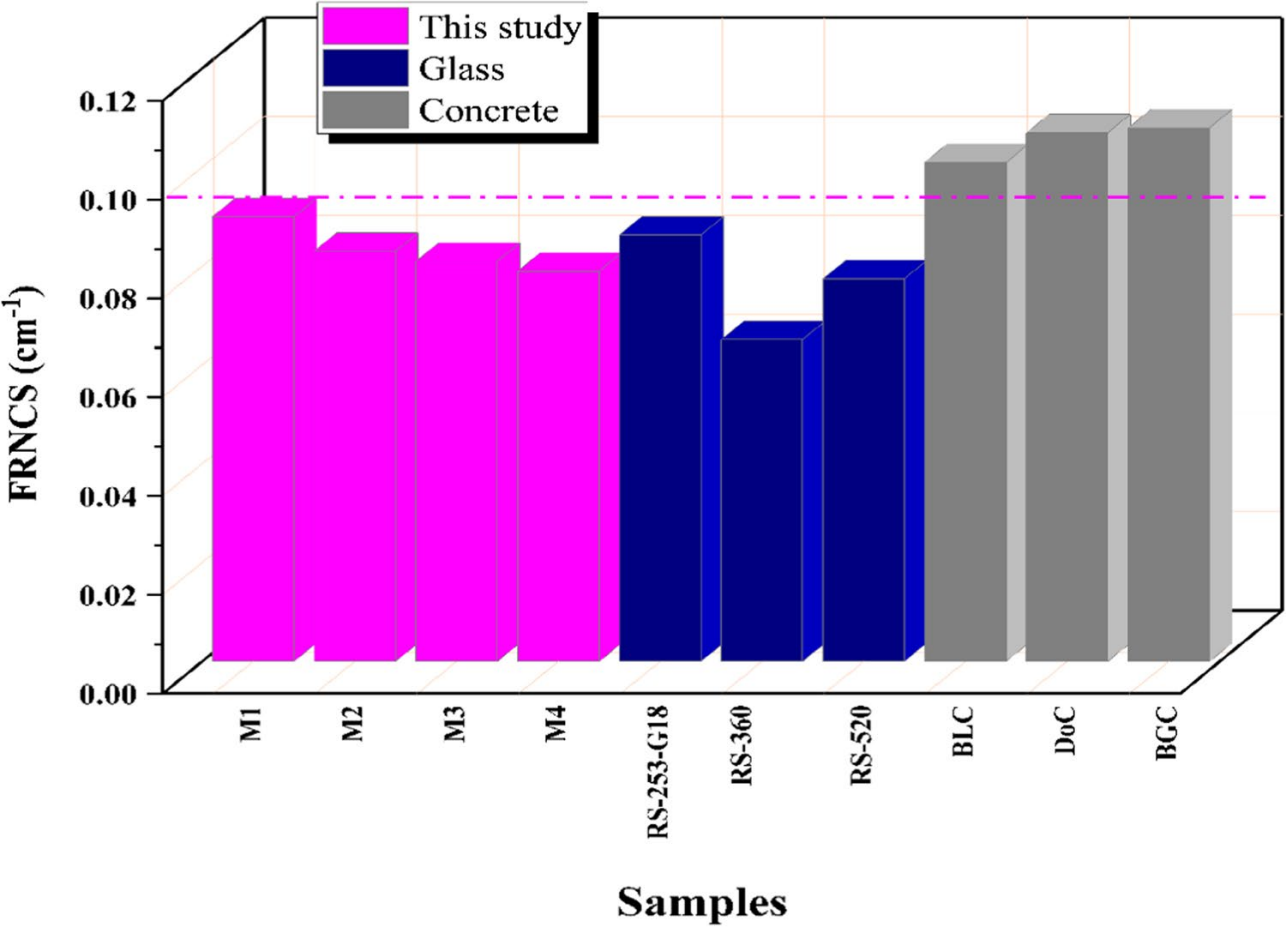
Comparison of the fast neutron removal cross-section (FRNCS) for the prepared M-X glass samples and commercial glass and concrete samples.

Figure 13 shows the fast neutrons removal cross-section (FRNCS) for the four prepared M-X glass samples, which were 0.090, 0.083, 0.081, and 0.079 cm^−1^, for M-X glasses where X = 1, 2, 3, and 4, respectively. Additionally, the FRNCS of the created M-X samples were compared to that of commercial glass samples, namely RS-253-G18, RS-520, and RS-360. Furthermore, three commercial concrete compounds, namely the concrete mix (DoC), goethite + boron carbide concrete mix (BGC), and limonite/sand concrete (BLC), were also included in the comparison^64^.

**Fig. 14:**
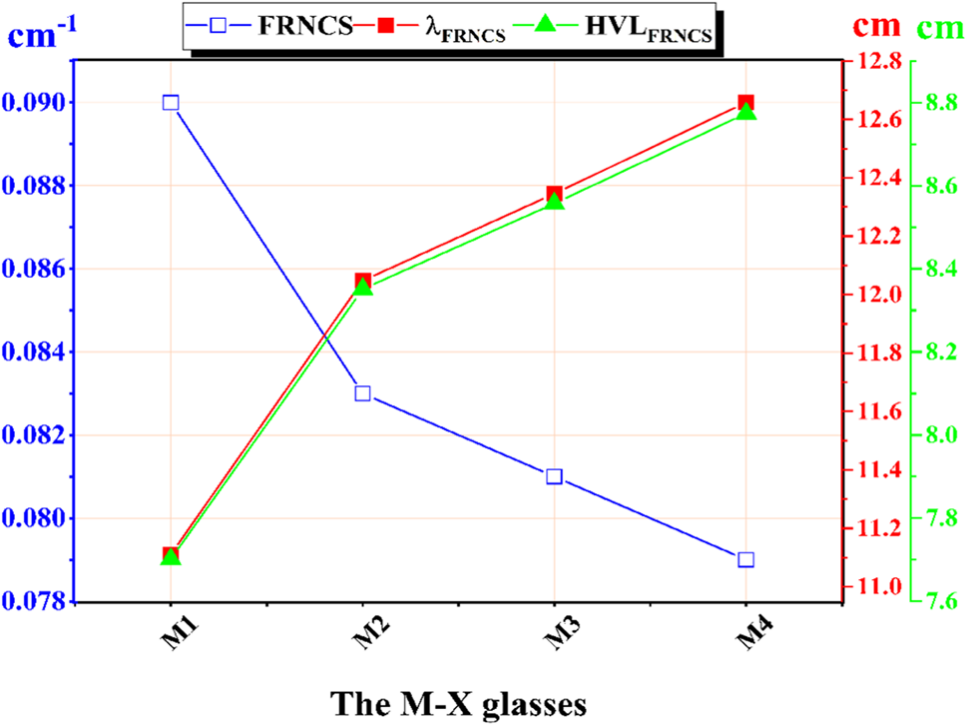
The fast neutron removal cross-section (FRNCS), the half value layer (HVL_FRNCS_), and the relaxation length (λ_FRNCS_) for the prepared M-X glass samples.

Figure 14 also displays the HVL_FRNCS_ and λ_FRNCS_ for the prepared M-X glass sample. Considering the simulated FRNCS values, the HVL_FRNCS_ and λ_FRNCS_ values for the M1 glass sample were the lowest.

## Conclusion

In this work we tried to enhance the optical and structural properties of the shielding glass by adding some dopant. Though the additives result in an enhancement of the structural and optical properties of the doped glasses, the base glass still had a tiny better radiation shielding features. This can be an indication that the doped glass would have a longer live time as a radiation shielding material, owing to its better structural properties, as observed from its optical studies. A slight modulation in the intensity of the preexisting peaks was seen, suggesting the glass structure’s resilience to radiation. This was particularly evident in the case of M4, where the curves remained virtually unchanged, both prior to and after irradiation. The data indicate that the inclusion of Dy, Ce, and Dy-Ce results in a decrease in the energy gap values. This has the potential to lead to the creation of defects that can effectively capture more free electrons, hence raising the likelihood of formation of non-bridging oxygens (NBOs) and enhancing the optical properties. Typically, when exposed to 75 kGy gamma irradiation, there is a correlation between an increase in the energy gap value and a decrease in non-bridging oxygen. Consequently, this leads to an increase in the compactness of the structure due to the higher concentration of bridging oxygen. In the case of incorporation of Ce^3+^, peaks appear at 195 nm for borate units and 225 nm for Ce^3+^ only for 4 F→5d transition (^2^F_5/2_→^5^D_1_) and at 360 nm broadened has been observed with higher intensity to be at 393 nm due to overlapping of two peaks for Ce^3+^ and Ce^4+^ in the UV region due to the 4f/5d transition of Ce^3+^ ions beside the Ce^4+^/Ce^3+^ charge transfer absorption peak of Ce^4+^ ions. By adding Dy; several peaks are observed at 825, 902, 1095, 1275, and 1684 nm, corresponding to the transition from ^6^H_15/2_ ground state to the transition states ^6^F_5/2_, ^6^F_7/2_, ^6^F_9/2_, ^6^F_11/2_ and ^6^H_11/2_; respectively. These modified glasses exhibited optical properties in response to the radiations. The M4 sample exhibited the most substantial difference in energy gap values, as evidenced by the higher conversion from NBO to BO, which is indicative of a more compact structure, as determined by the energy gap difference between before and after irradiation. This suggests that the M4 sample is more resistant to radiation-induced degradation, which improves its structural stability. Additionally, the increase in E_g_ values is significantly influenced by gamma rays. Consequently, it is possible to demonstrate that the conversion of non-bridging oxygen to bridging oxygen results in the repair of flaws in glass samples when they are exposed to gamma radiation. The number of defects within the glass matrix undergoes regeneration as the radiation exposure increases. This work also inspects the γ-rays and neutron protection features of the lead borate glasses without and with different dopants. The linear attenuation coefficient order was M1 > M2 > M3 > M4. The prepared samples M1, M2, M3, and M4 gave Z_ef_ values within the range of 79.631–49.007, 78.920–48.966, 78.898–49.062, and 78.197–48.704, respectively. The FRNCS values were 0.090, 0.083, 0.081, and 0.079 cm^−1^ for samples M1, M2, M3, and M4, respectively.

## Data Availability

The datasets used and/or analysed during the current study available from the corresponding author on reasonable request.
